# The Dangers of Treating Gonorrhœa with Simple Astringents

**Published:** 1897-12

**Authors:** F. G. Moehlau

**Affiliations:** Buffalo, N. Y.


### Selections and Abstracts.

### THE DANGERS OF TREATING GONORRHŒA WITH SIMPLE ASTRINGENTS.

By F. G. MOEHLAU, M. D., Buffalo, N. Y.

I have undertaken to investigate chronic diseases of the genito-urinary tract in thirty cases, and I think it will be of interest to many to learn of the result obtained.

The effect of syphilitic infection upon the general constitution of man, and its effect upon woman and the offspring, are constantly studied, and valuable observations have frequently been made. A good deal is known about the disease but so far the disease-producer has slipped discovery. But the remedial agents to battle the symptoms of the disease are most valuable and effective.

By careful observation we must come to the conclusion that gonorrhœa is doing more harm to mankind than syphilis. Of gonorrhœa we are aware of what produces the disease, i. e., the cause is definitely established by the presence of the gonococacus, discovered by Neiser.

Syphilitic infection renders the person immune for the future, though there are exceptions to the rule.

Gonorrhœa gives no immunity to succeeding infections, and of the remedial agents known and employed in the treatment none have proven specific, yet it is a fact that most men once infected with the disease, only the smaller number are definitely cured, which is certainly true of the older way of treatment, i. e., with the simple astringents as Plumbi, Acet. and Zinc Sulph.

The bad result obtained in treatment is much to be attributed to the difficulty to reach the affected parts, but more to the negligence of the patient. But, on account of the severe pain, great allowance has to be made for this.

The ignorance of the average "Clap Doctor" is the most harmful factor to the suffering community.

It seems wrong to teach physiology in public schools and most carefully exclude all reference to the genito-urinary tract. The pathology of alcoholism is included in such teaching, but in a most misleading way. Why not let the scholar be instructed about urethral diseases? It certainly would be a benefit to the community at large, and would do more good than what these wrong impressions about alcohol accomplish.

There would be much less of the nerve-destroying self-abuse, and in some respects act as a safeguard against prostitution.

To teach the physiology of the sexual organs in a proper way would do a great deal of self-protection to the ignorant youth. Unlimited is the number of innocent women who have to suffer for the errors of the husband; this mostly on account of ignorance. The physician called upon to treat those errors ought to do all in his power to enlighten the people. He is the one to be blamed for the indefinite amount of harm done, who is to treat a patient for gonorrhœa by making false promises, and gives false impressions to the sufferer, and who treats a patient and makes him believe that the stopping of the discharge means cure to the patient. Once the gonococcus has gained entrance to the urethra, it aims to progress and to multiply, from the mucosa it invades the submucous structure and proceeds, if not arrested, to invade the prostate, epidermis, testicle, bladder, ureters and kidneys. Other organs have no safeguard against such invasion.

A period of rest may succeed the most acute outbreak, to cause a renewed attack by slight provocation in any part of the body, as arthritis, pericarditis, even meningitis may have the gonococcus as the disease producer, once it has gained entrance to the human body.

To refer to the use of simple astringents in the treatment of gonorrhœa, if we consider the final result of their use we become cautious in their applications, as we know that even a weak solution causes a shriveling up of the mucosa, a contracting, and thus closing the orifices of the many ducts along the urethral canal, retaining the disease-producing organism in their lumen, not preventing its proliferation and the production of unknown quantities of toxines to be taken up by the lymph and blood stream, which definitely accounts for the fever and chills in an acute, subacute or even the chronic conditions of gonorrhœa, which, as is frequently observed, to resemble malarial fever. Treating gonorrhœa with simple astringents, we finally accomplish the arrest of the discharge, but the gonococcus is still in existence in the deeper layers of the lining of the urethral canal.

I have collected twenty cases, varying from five to thirty years since the primary infection, Tripperfæden not being present at all in seven of the cases. But an injection of 1:1000 of nitrate of silver produced a discharge in which the presence of gonococcus could be demonstrated beyond doubt.

The cases referred to had been treated by an injection of simple astringents and alkaline powders and waters. Under the existence of such circumstances the blood and nervous system must necessarily suffer severely, and in fact I find that patients in such condition present a chlorotic appearance, evidently due to the c[illegible]nic uræmia. The strength of the toxines produced by the gonococci varies greatly; there is more or less phagocytosis going on in all tissues.

The prostate, especially, seems to constitute an ideal storage place for the disease. In several of such cases I have observed by close questioning of the patient, that it needs little provocation to produce the discharge which will disappear again without interference, and to appear anew when irritated sufficiently.

The pelvic disease in women produced by such conditions in the husband's urethra or prostate are innumerable, 90 per cent. of all pelvic diseases we have to attribute to such state of circumstances. It takes years in many instances to give evidence of symptoms of the inflammatory conditions existing in the pelvic organs of the innocent woman. Sterility after the first accouchment, due to the inflamed uterus, is frequent. Abortion after the first child, where the uterus has been sound until then, are not rare, and poorly developed, anæmic or even rickety offsprings call strong and vigorous people their parents. It is natural, that where blood mixed with toxines of various sorts is used in the manufacture of the sperma, perhaps conception of an ovum of the same quality can produce no healthy nor strong child when mature, especially if the intra-uterine nourishment is from blood saturated with toxines of varying strength. The osmosis of those well mixed and diluted toxines, the placenta, finally, is unable to prevent. Just like alkaloids pass to the fœtus and exert their due and noxious influence, as has been demonstrated with opiates. With such facts before us it ought to be the aim of every practitioner to do the utmost possible to cause the patient to grasp the condition he is in, and to understand what it means to be cured of gonorrhœa. At the same time I take opportunity to refer to a condition produced in middle age, which seems to be due to the errors of youth. This is an obstinate back ache, which is promptly relieved by letting the sufferer wear a well-fitting suspensory; better yet, I am satisfied by the results obtained by the good adjusting of a T bandage drawn up well, so as to prevent the testicles from exerting traction upon the attachment part of the cords. This at the same time I find to exert a reducing influence upon the enlarged prostate.—*Amer. Jour. of Derm. and G.-U. Diseases.*

